# Ferritin Reference Curves and Optimal Curves in Preadolescent Children

**DOI:** 10.1001/jamanetworkopen.2026.13041

**Published:** 2026-05-15

**Authors:** Vid Bijelić, Franco Momoli, Mira Liebman, Beth K. Potter, Cornelia M. Borkhoff, Catherine S. Birken, Jonathon L. Maguire, Patricia C. Parkin, Jemila S. Hamid

**Affiliations:** 1 School of Epidemiology and Public Health, Faculty of Medicine, University of Ottawa, Ottawa, Ontario, Canada; 2 Clinical Research Unit, Children’s Hospital of Eastern Ontario, Ottawa, Ontario, Canada; 3 Department of Pediatrics, Children’s Hospital of Eastern Ontario, Ottawa, Canada; 4 Institute of Health Policy, Management and Evaluation, University of Toronto, Toronto, Ontario, Canada; 5 Child Health Evaluative Sciences, Hospital for Sick Children Research Institute, Toronto, Ontario, Canada; 6 Department of Pediatrics, Faculty of Medicine, University of Toronto, Toronto, Ontario, Canada; 7 MAP Centre for Urban Health Solutions, Unity Health Toronto, Toronto, Ontario, Canada; 8 Department of Mathematics and Statistics, University of Ottawa, Ottawa, Ontario, Canada

## Abstract

**Importance:**

Ferritin levels are commonly used to assess iron status. For interpretation, clinicians traditionally rely on age-partitioned reference intervals (RIs), which report 2.5th (lower) and 97.5th (upper) percentiles.

**Objectives:**

To estimate sex-specific continuous ferritin reference curves (RCs) and optimal curves (OCs) for preadolescents; to create age- and sex-specific RIs and optimal intervals (OIs); to interpret estimates alongside ferritin thresholds for iron deficiency; and to develop an interactive web-based computational tool.

**Design, Setting, and Participants:**

This cross-sectional analysis used data from a longitudinal cohort study. Participants included 4935 children aged 2 weeks to 10 years attending scheduled primary care health supervision visits from June 3, 2008, to February 26, 2020, in Toronto, Ontario, Canada. Data were analyzed from October 16, 2024, to December 17, 2025.

**Exposure:**

Blood samples were collected and analyzed for levels of ferritin, hemoglobin, and C-reactive protein. Parents completed a questionnaire collecting optimality criteria for iron status.

**Main Outcomes and Measures:**

Sex-specific RCs were estimated using generalized additive models for location, scale, and shape. OCs were derived from a subsample of participants meeting predefined optimality criteria, using multiple imputation to address missing data. RCs and OCs were interpreted in the context of the World Health Organization threshold (<12 ng/mL) and the proposed American Society of Hematology (ASH) threshold (≤20 ng/mL).

**Results:**

RCs were estimated for 4935 children (2613 [52.9%] male) aged 2 weeks to 10 years (median age, 37 [IQR, 18-62] months). OCs were estimated from a subset of 3630 children (1909 [52.6%] male). Curves were highest in early infancy, declined sharply at 1.5 years of age (lower limit, 5-6 ng/mL), then gradually increased at 9 to 10 years of age (lower limit, 15-17 ng/mL). RCs and OCs showed similar trajectories, with slightly higher limits for OCs. The proportion of preadolescent children in the optimal population who would be classified as having iron deficiency in existing (World Health Organization) and proposed thresholds differed with age. Using the ASH threshold in this cohort, 54.7% of males and 37.0% of females would be considered to have iron deficiency at 18 months.

**Conclusions and Relevance:**

In this cross-sectional study, ferritin RCs and OCs were estimated in a low-risk population of preadolescent children. The lower limits of curves and intervals were substantially lower than physiologically based thresholds to define iron deficiency. These findings further inform the current discussion regarding the distributional vs physiologic approaches to interpreting ferritin measurements and guiding clinical decision-making.

## Key Points

**Question:**

What are the lower and upper limits of the ferritin reference curves for preadolescent children, and are these curves associated with physiologically based iron deficiency thresholds?

**Findings:**

In this cross-sectional study of 4935 children, the limits were highest in early infancy, declined at 1.5 years of age (lower limit, 5-6 ng/mL), then gradually increased at 9 to 10 years of age (lower limit, 15-17 ng/mL). From 3 months to 10 years of age, the lower limit of ferritin level was below the physiologically based threshold of 20 ng/mL or less.

**Meaning:**

These findings suggest that reference curves, which provide lower and upper limits continuously across age, may improve clinical utility, despite the lower limits being below the proposed threshold for identifying iron deficiency in pediatrics.

## Teaser

This cohort study estimates reference curves and optimal curves for serum ferritin measurements among children aged 2 weeks to 10 years in Canada to inform the discussion regarding the distributional vs physiologic approaches to interpreting ferritin measurements.

## Introduction

Ferritin levels, measured in serum or plasma, are commonly used in clinical practice to assess iron stores, with low levels indicating iron deficiency and high levels indicating iron overload or inflammation.^1,2^ In pediatrics, iron deficiency prevalence is highest in early childhood for both sexes and for adolescent females. Iron deficiency is the leading cause of anemia and is associated with neurodevelopmental impairment, fatigue, and decreased concentration.^1^ Iron overload is much less common in children, but high levels of ferritin are associated with inflammation, malignant neoplastic disease, and obesity.^1^

To interpret ferritin levels, clinicians rely on reference intervals (RIs) developed following Clinical Laboratory Standards Institute guidelines^3,4^ from samples of healthy reference populations with adequate sample sizes.^3^ RIs are defined by the 2.5th and 97.5th percentiles corresponding to lower and upper RI limits, with 90% CIs for each limit. Sex- and age-specific partitions may be required, particularly in the pediatric population. Our systematic review of pediatric RIs for ferritin^5^ identified different age partitions across studies and significant heterogeneity among reported RIs, limiting our ability to perform a meta-analysis to generate reliable pooled estimates.

Reference curves (RCs), which model biomarkers as a continuous function of age, offer an alternative to age-partitioned RIs, which is especially important in pediatrics when considering physiologic changes from infancy through adolescence.^6,7^ The method is similar to the approach taken for developing the World Health Organization (WHO) growth standards.^8^ In our systematic review,^5^ we identified 4 studies on pediatric RCs for ferritin,^9,10,11,12^ and only 1 provided CIs for inclusion in our meta-analysis.^9^

An alternative approach, described in anthropometric research, involves selecting a subsample of individuals free from health conditions and environmental or socioeconomic risk factors related to the biomarker of interest.^13,14^ Our systematic review^5^ found no studies that excluded children at risk of iron deficiency or anemia, suggesting existing estimates may not reflect optimal iron health. Applying optimality criteria may provide more clinically meaningful reference limits.

Emerging evidence suggests that reliance on the distribution-based lower limit of the ferritin RI (2.5th percentile) leads to underdiagnosis of iron deficiency in children and adults, with experts advocating for physiologically based thresholds (clinical decision limits).^15,16,17,18,19,20,21,22,23,24,25,26,27,28^ The WHO recommends a ferritin threshold of less than 12 ng/mL (to convert to µg/L, multiply by 1) to define iron deficiency for children younger than 5 years and less than 15 ng/mL for those 5 years and older, largely based on consensus.^1^ The 2025 American Society of Hematology (ASH) draft recommendations suggest a ferritin threshold of 20 ng/mL or less for children aged 9 months to 4 years, based on a single pediatric study comparing ferritin with bone marrow iron levels.^29^ Using these thresholds rather than the lower RI limit will substantially increase the number of children considered to have iron deficiency.^21^ Our objectives were to estimate sex-specific ferritin RCs for preadolescent children, estimate optimal curves (OCs) using predefined optimality criteria, create age- and sex-specific RIs and optimal intervals (OIs), contextualize our estimates alongside WHO and proposed ASH iron deficiency thresholds, and develop an interactive web-based computational and graphical tool.

## Methods

### Population and Study Design

We used cross-sectional data from healthy children who participated in a longitudinal cohort study from a primary care research network in Toronto, Ontario, Canada.^30^ Children were recruited at any scheduled health supervision visits at 2 weeks or 2, 4, 6, 9, 12, 15, or 18 months of age and then annually to 5 years of age. Parents completed questionnaires covering demographic characteristics and medical history, including birthweight, gestational age, and household income. Trained research assistants in participating clinics collected anthropometric measures and blood samples at multiple visits, with blood sample collection being optional. Pre–COVID-19 data (June 3, 2008, to February 26, 2020) from children aged 2 weeks to 10 years were included. Children with health conditions affecting growth, acute or chronic conditions (other than asthma and high-functioning autism), or severe developmental delay and those with families unable to communicate in English were excluded.^30^ Blood samples were collected in serum-separator and EDTA tubes, transported at room temperature on the same day to the accredited research laboratory at Mount Sinai Services, Toronto, and analyzed within 4 to 6 hours for ferritin, hemoglobin, and C-reactive protein (CRP) levels on automated hematology analyzers (XN-9000 systems [Sysmex] and Modular E170 analyzer[Roche Diagnostics]). ^30^

The Research Ethics Boards at the Hospital for Sick Children and St Michael’s Hospital, Toronto, granted approval, and written informed consent was obtained from parents of participating children. We followed the Strengthening the Reporting of Observational Studies in Epidemiology (STROBE) reporting guideline.

### Optimal Curve Subgroup

To create a subgroup at low risk of iron deficiency, we used prespecified optimality criteria obtained from previous research^31,32,33,34^ and recommendations^35,36,37^ regarding factors associated with iron status. Data were excluded from children with anemia (hemoglobin level for those aged 6-23 months, <10.5 g/dL; aged 24-59 months, <11.0 g/dL; and ≥60 months or older, <11.5 g/dL [to convert hemoglobin to g/L, multiply by 10]),^38^ living in low-income households (<$42 000 Canadian [US $30 230]),^39^ and/or having underweight or overweight (above or below a body mass index [BMI; calculated as weight in kilograms divided by height in meters squared] *z* score of ±1.96 × SD, respectively).^33^ BMI was calculated from measured height or length and weight standardized for age and sex using WHO growth standards.^30,33,40^ The income threshold approximated the mean Statistics Canada low income cutoff before tax for a family of 4 residing in urban area with a population greater than 100 000 residents between 2008 and 2020.^39^ We excluded data from children younger than 2 years if born prematurely (gestational age <37 weeks) or with low birth weight (<2500 g)^37^ or if the CRP level was greater than 0.5 mg/dL [to convert to mg/L, multiply by 10]), suggesting acute systemic inflammation that can falsely elevate ferritin levels.^41^

### Statistical Analysis

Data were analyzed from October 16, 2024, to December 17, 2025. Some children had repeated measurements; therefore, we selected 1 ferritin measurement per child, prioritizing observations from ages with sparse data (termed *lean pick*).^5^ We estimated sex-specific RCs and OCs using the same analytic methods. RCs included the overall cohort. OCs included the subgroup meeting optimality criteria. The generalized additive models for location, scale, and shape (GAMLSS) were used to model the age-dependent distribution of ferritin separately for males and females. We applied the Box-Cox-t transformation that accommodates skewed and heavy-tailed distributions and selected the log-link function based on generalized Akaike information criteria.^42^ We used the gamlss package in R, version 5.4-22 (R Project for Statistical Computing).^43,44^ Parameters of distributions were estimated as a smooth function of age using penalized B splines.^45^ Outliers were identified using Dunn-Smyth residuals (±3 *z* scores) and removed.^46^ The model was refitted to the remaining observations. This process was iteratively repeated until no additional outliers were identified. We estimated 90% CIs^3^ for the fitted percentile curves using the nonparametric bootstrap method (200 bootstrap samples).

We estimated the 5th, 10th, and 20th percentile curves to allow comparisons with WHO and ASH iron deficiency thresholds for children aged 2 weeks to 5 years.^47^ Informed by previous studies,^10,48^ we derived age- and sex-specific RIs and OIs from the RCs and OCs by estimating ferritin values at the 2.5th and 97.5th percentiles using estimates from the fitted GAMLSS. Lower and upper limits were calculated for each month of age and for previously proposed standardized age intervals^5^: 3-month intervals for children younger than 3 years, and 1-year intervals afterward.

Using the Optimized Reference Assessment for Clinical Laboratory Evaluation for Ferritin (ORACLE-FER) Shiny application,^49^ we developed a web-based computational and graphical tool to visualize estimated curves, to compare with WHO and ASH thresholds, and to allow interactive age- and sex-specific estimation of RIs and OIs based on fitted models. Statistical analyses were performed using R, version 4.4.1.^44^ RCs were compared descriptively through visual inspection across the age range. No formal statistical tests were performed.

RCs were estimated using data from participants with at least 1 ferritin level measurement, maximizing our sample size for younger children using a lean pick approach. For OCs, we used multiple imputations by chained equations to handle missing data for variables used to define the optimality criteria.^50,51^ In our multiple imputation model, we incorporated available repeated measurements and both time-varying and time-invariant variables. Restricted cubic splines were used to model nonlinear relationships. Multiple imputation models included age in months and ferritin (both with knot terms) level, hemoglobin level, household income (defined as the middle of the reporting interval), BMI *z* score, prematurity, and low birthweight.

To manage missing ferritin data when imputing optimality criteria variables, our primary OC estimation analysis followed the multiple-imputation-then-deletion (MID) approach under the missing-at-random assumption.^52^ Initially, we relied on the full dataset, including participants with and without ferritin values, to impute data on optimality criteria. We then excluded participants with no ferritin values or with missing CRP values to estimate OCs. Two sensitivity analyses were conducted to assess robustness of the findings to this strategy for managing missing data: deletion-then-multiple-imputation (DMI) approach, where records with missing ferritin values were excluded prior to imputation; and complete-case analysis, where only participants with observed optimality criteria were included in the OC estimation. Eighty-one imputed datasets were generated in the primary MID approach and 27 in the DMI approach.^53^ For each approach, results were combined using Rubin rules.^50^ Imputation was implemented using the mice package in R, version 3.17.0, the miceadds package in R, version 3.17.44,^51^ and the rms package for restricted-cubic-splines in R, version 6.8-2.^54^

## Results

### Study Characteristics

Of 11 802 children enrolled, blood samples were obtained and analyzed, including ferritin value measurements, for 4935 children (2322 [47.1%] female and 2613 [52.9%] male) aged 2 weeks to 10 years (median age, 37 [IQR, 18-62] months); we used these values to estimate sex-specific RCs (Table 1). Children in the cohort with and without ferritin measurements appeared to have similar characteristics. Sample size by age is shown in eTable 1 in Supplement 1.

**Table 1.** Characteristics of Study Participants

Characteristics	All (N = 11 802)^a^	Participants with ferritin values (n = 4935)^b^	Participants with ferritin values, imputation^c^	
			All (n = 4935)	Optimality criteria (n = 3630)^d^
Age, median (IQR), mo	34 (11-69)	37 (18-62)	37 (18-62)	40 (21-64)
Sex, No. (%)				
Female	5672 (48.1)	2322 (47.1)	2322 (47.1)	1721 (47.4)
Male	6130 (51.9)	2613 (52.9)	2613 (52.9)	1909 (52.6)
Ferritin level, median (IQR), ng/mL	30 (21-43)	29 (20-41)	29 (20-41)	29 (21-40)
Missing, No. (%)	9384 (79.5)	0	0	0
CRP level, median (IQR), mg/dL	0.03 (0.02-0.07)	0.03 (0.02-0.06)	0.03 (0.02-0.06)	0.02 (0.02-0.05)
No. missing	9121	0	0	0
Low-income cutoff, No. (%)				
Above	7688 (89.1)	3985 (90.5)	4464 (90.5)	3630 (100)
Below	941 (10.9)	417 (9.5)	471 (9.5)	0
No. Missing	3173	533	0	0
Prematurity, No. (%)^e^				
Not premature	9850 (95.9)	4555 (96.0)	4734 (95.9)	3630 (100)
Premature	417 (4.1)	189 (4.0)	201 (4.1)	0
No. missing	1535	191	0	0
Low birthweight, No. (%)^e^				
Absent	10 285 (96.2)	4678 (96.3)	4746 (96.2)	3630 (100)
Present	409 (3.8)	181 (3.7)	189 (3.8)	0
No. missing	1108	76	0	0
Overweight or underweight, No. (%)^f^				
Absent	10 313 (90.6)	4447 (92.0)	4539 (92.0)	3630 (100)
Present	1071 (9.4)	385 (8.0)	396 (8.0)	0
No. missing	418	103	0	0
Anemia, No. (%)				
Absent	3650 (95.6)	4189 (92.5)	4569 (92.6)	3630 (100)
Present	169 (4.4)	341 (7.5)	366 (7.4)	0
No. missing	7983	405	0	0

Abbreviation: CRP, C-reactive protein.

SI conversion factors: To convert CRP to mg/L, multiply by 10; to convert ferritin to µg/L, multiply by 1.

^a^ Cross-sectional sample was derived from the full TARGet Kids! longitudinal dataset using the lean-pick method.

^b^ Sample was used for reference curve estimation.

^c^ Missing data (not included in denominator for other category) for optimality criteria variables imputed with multiple imputation then deletion approach (see Methods).

^d^ Sample for optimal curve estimation.

^e^ Applicable when ferritin level was measured in first 24 months of life.

^f^ Indicates body mass index (BMI) *z* score ± 1.96 × SD, where BMI *z* score is relative to World Health Organization reference population adjusted for age and sex.

Sex-specific OCs were estimated using a subsample of 3630 children (1909 [52.6%] male and 1721 [47.4%] female) meeting the optimality criteria. Participants in a nonoptimal category ranged from 189 (3.8%) with low birthweight to 471 (9.5%) with household income below the low-income cutoff when each variable was considered separately, with 1283 (26.0%) excluded due to being in a nonoptimal category for 1 or more of the criteria (Table 1). Eight males and 3 females were considered extreme outlier observations and were therefore removed from both the RC and OC analyses.

### Reference and Optimal Curves

**Figure 1.**
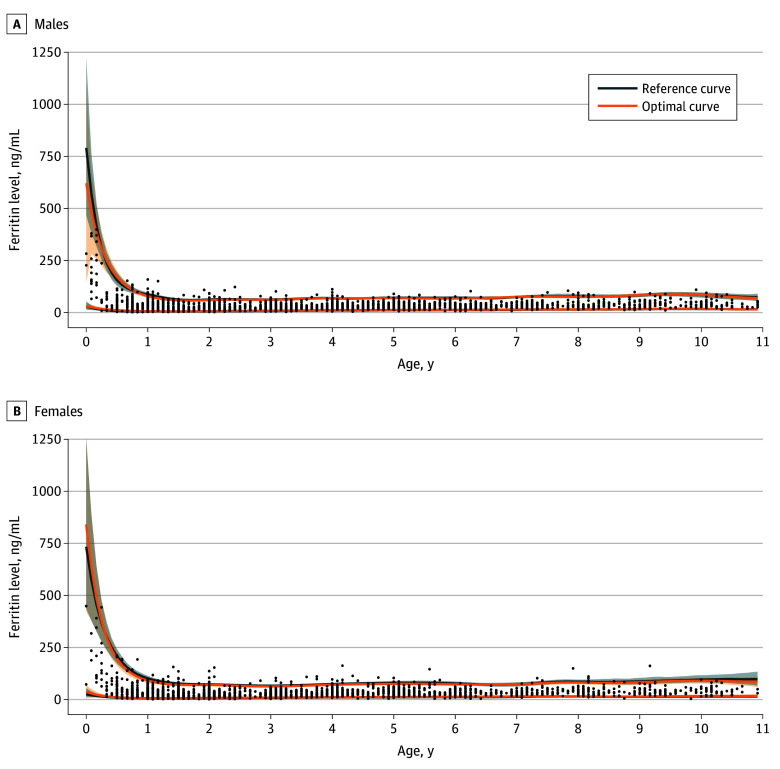
Line Graphs Showing Ferritin Reference Curves and Optimal Curves for Children Aged 2 Weeks to 10 Years The actual measurements of ferritin levels from the 4935 children are presented using scatter plots for 2613 males and 2322 females. Dots indicate individual measurements; shaded areas indicate 90% CIs.

For males and females aged 2 weeks to 10 years, RCs and OCs showed a similar pattern with respect to age, with higher variation in older females (Figure 1). Although the OC lower limits were consistently above the RC lower limits, the 2 curves generally overlapped. Both lower and upper limits were high in early infancy and sharply declined during the first year of life, reaching a minimum at approximately 1.0 to 1.5 years of age, with the lower limit reaching approximately 5 to 6 ng/mL. Both curves gradually increased after the second year of life, reaching a maximum at approximately 9 to 10 years, with the lower limit reaching approximately 15 to 17 ng/mL. The lowest ferritin values at 18 months of age were 5.9 ng/mL (90% CI, 5.3-6.5 ng/mL) from the OC for males and 6.8 ng/mL (90% CI, 6.1-7.5 ng/mL) from the OC for females.

### Iron Deficiency Thresholds

**Figure 2.**
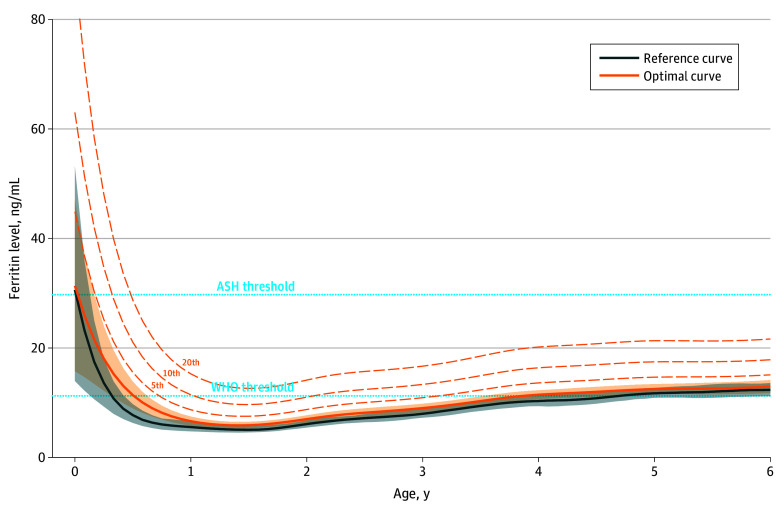
Line Graphs Showing Lower Limits of Ferritin Reference Curves and Optimal Curves for Males Aged 2 Weeks to 5 Years The World Health Organization (WHO) and American Society of Hematology (ASH) ferritin thresholds for iron deficiency are also presented. Additional optimality percentile curves are represented by dashed curves. Shaded areas indicate 90% CIs.

**Figure 3.**
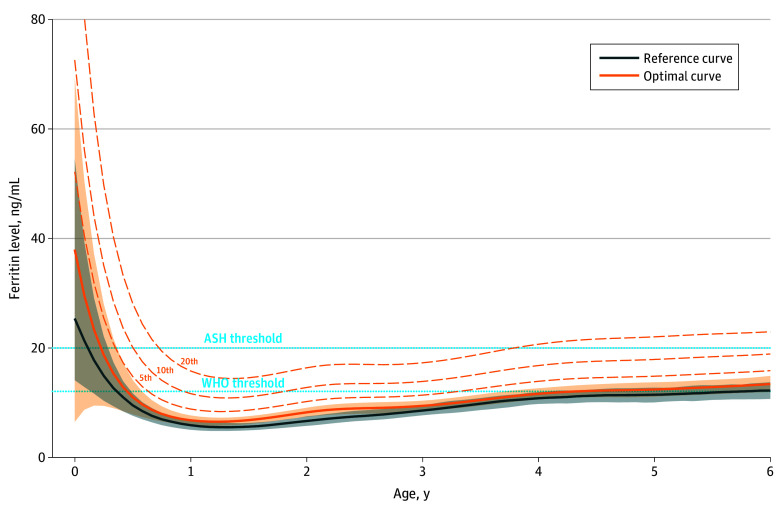
Line Graphs Showing Lower Limits of Ferritin Reference Curves and Optimal Curves for Females Aged 2 Weeks to 5 Years The World Health Organization (WHO) and American Society of Hematology (ASH) ferritin thresholds for iron deficiency are also presented. Additional optimality percentile curves are represented by dashed curves. Shaded areas indicate 90% CIs.

Age-specific 5th, 10th, and 20th percentile thresholds as well as WHO and ASH thresholds for the optimal populations of children aged 2 weeks to 5 years are shown in Figure 2 (males) and Figure 3 (females). For both males and females, the WHO threshold generally fell between the 5th and 10th percentile of our OCs, but with fluctuation over age. The ASH threshold exceeded the 20th percentile between 9 months and 3.5 to 4.0 years of age. The proportion of children in the optimal population who would be classified with iron deficiency for each threshold differed by child age. For example, at 18 months of age, the proportion for males and females would be 19.7% and 15.1%, respectively, using the WHO threshold, and 54.7% and 37.0%, respectively, using the ASH threshold.

### Reference Intervals

Table 2 provides RIs and OIs (lower and upper limits) with the corresponding 90% CIs for males and females, based on 3-month age intervals for children younger than 3 years and yearly intervals for those 3 years or older. Results using monthly age intervals are presented in eTable 2 in Supplement 1.

**Table 2.** Age- and Sex-Specific Reference and Optimal Intervals for Ferritin Values

Age group	Ferritin level, ng/mL			
	Lower limit (90% CI)		Upper limit (90% CI)	
	Optimal	Reference	Optimal	Reference
**Males**				
2 wk to 2 mo	26.2 (14.6-37.9)	23.7 (12.3-38.0)	496.0 (231.1-760.9)	592.0 (389.0-836.0)
3-5 mo	15.5 (11.0-19.9)	11.1 (8.1-14.4)	252.9 (195.4-310.4)	245.7 (203.4-298.4)
6-8 mo	10.1 (8.2-12.1)	7.0 (5.8-8.7)	146.4 (124.2-168.5)	135.7 (113.0-164.2)
9-11 mo	7.6 (6.5-8.7)	5.8 (5.0-6.9)	97.5 (86.1-109.0)	99.0 (88.8-112.9)
12-14 mo	6.3 (5.6-7.1)	5.4 (4.7-6.5)	73.8 (66.5-81.1)	81.3 (73.9-90.6)
15-17 mo	5.9 (5.2-6.5)	5.1 (4.6-5.8)	62.4 (56.9-67.9)	68.5 (63.3-75.0)
18-20 mo	6.0 (5.4-6.6)	5.1 (4.6-5.8)	58.1 (53.3-63.0)	61.4 (56.5-67.2)
21-23 mo	6.5 (5.9-7.1)	5.6 (5.0 -6.2)	58.5 (53.9-63.2)	61.0 (56.9-66.3)
24-26 mo	7.2 (6.5-7.9)	6.3 (5.6-7.0)	60.7 (55.7-65.7)	63.3 (58.9-69.3)
27-29 mo	7.9 (7.1-8.6)	6.9 (6.2-7.5)	61.8 (56.5-67.2)	63.9 (58.6-70.3)
30-32 mo	8.3 (7.5-9.1)	7.3 (6.5-8.1)	61.6 (56.2-67.0)	62.9 (56.9-69.3)
33-35 mo	8.7 (7.9-9.5)	7.6 (6.9-8.6)	61.4 (56.4-66.4)	62.4 (57.1-68.9)
3 to <4 y	10.2 (9.4-11)	9.2 (8.3-10.2)	65.1 (60.1-70.0)	66.1 (60.8-72.7)
4 to <5 y	11.9 (11.1-12.8)	10.9 (9.9-11.9)	69.3 (64.6-73.9)	69.0 (63.8-75.1)
5 to <6 y	12.6 (11.6-13.6)	12.0 (11.0-13.2)	68.3 (63.5-73.1)	71.3 (65.8-76.8)
6 to <7 y	13.7 (12.4-15.0)	12.6 (11.2-14.0)	70.5 (64.8-76.1)	70.5 (64.3-78.4)
7 to <8 y	15.3 (13.9-16.8)	14.5 (13.0-16.3)	76.8 (70.0-83.7)	77.8 (69.8-87.3)
8 to <9 y	15.8 (14.2-17.4)	15.2 (13.3-17.0)	78.8 (71.5-86.2)	78.7 (70.6-88.2)
9 to <10 y	17.9 (15.9-19.9)	17.3 (15.0-20.1)	86.9 (78.0-95.9)	86.9 (76.6-96.9)
10 to <11 y	16.6 (14.1-19.1)	16.5 (13.9-19.4)	76.7 (65.5-88.0)	78.6 (64.0-92.2)
**Females**				
2 wk to 2 mo	30.3 (8.2-52.3)	21.5 (12.9-41.0)	653.7 (378.2-929.3)	589.2 (382.1-928.3)
3-5 mo	15.6 (9.0-22.3)	12.9 (9.4-17.8)	303.8 (237.0-370.5)	305.6 (239.9-380.2)
6-8 mo	9.8 (7.4-12.2)	8.5 (7.0-10.3)	167.5 (140.8-194.2)	180.4 (153.1-208.0)
9-11 mo	7.5 (6.3-8.7)	6.6 (5.6-7.6)	111.3 (95.5-127.2)	122.7 (107.6-138.2)
12-14 mo	6.7 (5.8-7.5)	5.7 (4.9-6.5)	86.3 (75.9-96.7)	95.3 (85.9-106.2)
15-17 mo	6.6 (5.9-7.3)	5.5 (4.9-6.3)	75.2 (67.5-82.9)	81.7 (74.8-89.7)
18-20 mo	7.0 (6.3-7.7)	5.7 (5.1-6.4)	71.1 (64.3-77.9)	75.9 (68.8-82.1)
21-23 mo	7.7 (7.0-8.5)	6.3 (5.5-7)	70.4 (63.8-76.9)	73.6 (67.2-79.6)
24-26 mo	8.5 (7.6-9.3)	6.9 (5.9-7.7)	70.1 (63.3-76.9)	72.3 (65.8-78.9)
27-29 mo	8.9 (7.9-9.8)	7.3 (6.5-8.3)	68.2 (61.4-75)	70.1 (62.9-77.2)
30-32 mo	9.0 (8.0-10.1)	7.7 (6.9-8.7)	65.3 (58.8-71.9)	68.2 (60.2-75.1)
33-35 mo	9.2 (8.2-10.2)	8.2 (7.3-9.1)	63.6 (57.8-69.5)	66.9 (59.5-73.3)
3 to <4 y	10.4 (9.4-11.4)	9.7 (8.6-10.7)	66.6 (60.9-72.3)	69.6 (63.2-75.8)
4 to <5 y	12.1 (10.9-13.3)	11.2 (10.0-12.4)	73.9 (67.9-79.9)	77.3 (70.9-84.6)
5 to <6 y	12.8 (11.5-14.2)	11.8 (10.4-13.1)	75.3 (68.1-82.6)	80.1 (72.6-89.9)
6 to <7 y	14.1 (12.3-15.9)	12.4 (10.9-14.7)	72.6 (65.5-79.7)	73.8 (65.4-82.2)
7 to <8 y	15.8 (13.9-17.6)	14.6 (12.6-17.2)	78.8 (70-87.5)	81.3 (71.8-91.1)
8 to <9 y	14.7 (12.8-16.7)	14.0 (12.1-15.8)	81.4 (70.6-92.2)	85.4 (75.2-99.0)
9 to <10 y	15.8 (13.5-18.2)	14.0 (11.4-16.5)	88.0 (74.5-101.4)	91.3 (78.9-110.1)
10 to <11 y	17.6 (13.3-22.0)	15.1 (10.6-20.4)	88.5 (71.1-106)	97.8 (77.6-123.9)

SI conversion factor: To convert ferritin to µg/L, multiply by 1.

### Sensitivity Analysis

Sensitivity analyses using the DMI instead of MID strategy yielded no substantive differences in OCs for males and females (eFigures 1 and 2 and eTable 3 in Supplement 1). In a complete-case analysis, OC estimates for males were also similar but the MID approach yielded slightly narrower 90% CIs, particularly in infancy and late childhood (eFigures 3 and 4 in Supplement 1). In females, a complete-case analysis generated similar OC estimates to the MID approach except at 8 years and older, where complete case analysis estimates were more variable with notably wider 90% CIs, especially in the upper limit of OCs.

### Interactive Web-Based Tool

The ORACLE-FER Shiny application^49^ is an interactive web-based tool designed to support the interpretation of ferritin concentrations for pediatric populations based on our study findings. The application enables users to explore ferritin RIs generated from RCs and OIs generated from OCs for children aged 2 weeks to 10 years. The user can enter the child’s sex and birthdate or age to view age- and sex-specific RIs and OIs, to visualize the optimality zone (the area between the lower and upper OCs), and to compare values with the WHO and ASH thresholds. The application supports multiple age formats (custom ranges, WHO age categories, standardized intervals) and displays an individual child’s ferritin value relative to RCs and OCs.

## Discussion

In this cross-sectional study, we generated sex-specific ferritin RCs based on cross-sectional data from a healthy cohort of 4935 Canadian children aged 2 weeks to 10 years. We then applied prespecified optimality criteria to define a subpopulation at lower risk of iron deficiency to estimate sex-specific OCs. Given pronounced skewness of the distribution of ferritin measurements, we used GAMLSS, which accommodate asymmetry and enables simultaneous modeling of the distribution’s mean, variance, skewness, and kurtosis as smooth functions of age. Multiple imputation was used to handle missing data for optimality criteria. From RCs and OCs, we generated age- and sex-specific RIs and OIs with their corresponding 90% CIs. We developed a web-based tool that enables users to obtain individualized RI and OI estimates for ferritin levels based on a child’s exact age and sex and contextualizes the results with respect to WHO and ASH iron deficiency thresholds.

Our curves for ferritin values revealed a dynamic association with age in preadolescent children, with the lower limit reaching a minimum of 5 to 6 ng/mL at 1.5 years and a maximum of 15 to 17 ng/mL at 9 to 10 years. The similarity between ferritin RCs and OCs likely reflects the profile of the cohort, which was designed to represent a generally healthy population.^30^ Furthermore, while our optimality criteria aimed to create a low-risk subgroup, this is not equivalent to ideal iron status. Applying optimality criteria did not appear to substantially add incremental interpretive value.

Of 4 studies we identified that have developed pediatric ferritin RCs, 2 used quantile regression^9,10^ and 2 used the lambda-mu-sigma (LMS) method.^11,12^ Three of the 4 studies^9,10,12^ reported ferritin RCs among children younger than 4 years, and no studies reported values for children younger than 1 year. In our study, the lowest ferritin values at 18 months of age were 5.9 ng/mL (90% CI, 5.3-6.5 ng/mL) from the OC for males and 6.8 ng/mL (90% CI, 6.1-7.5 ng/mL) from the OC for females. By comparison, the single study^9^ that provided 90% CI reported 6.5 ng/mL (90% CI, 1.5-11.4 ng/mL) for males and 9.0 ng/mL (90% CI, 6.5-11.5 ng/mL) for females at 18 months of age. The other 2 studies^10,12^ that reported values at 18 months of age reported values of 2.5 and 7.1 ng/mL for males and 9.0 and 11.5 ng/mL for females. Across studies, the lower limit was lower for males compared with females, which may be due to more rapid growth in males.^55^

There is a developing consensus that the distribution-based lower RI limit (2.5th percentile) leads to underdiagnosis of iron deficiency.^29^ The ASH threshold of 20 ng/mL or less^29^ is based on a single diagnostic accuracy study comparing ferritin measurements with bone marrow iron values in 87 children in Malawi (aged 6-66 months) undergoing elective surgery.^56^ More recent studies using a physiologically based approach comparing ferritin with hemoglobin support this threshold.^15,16,17,18,19,20^ Our findings suggest that applying a threshold of 20 ng/mL or less (rather than the lower RI limit or WHO threshold) will substantially increase the proportion of children diagnosed with iron deficiency. This is especially true for children younger than 3 years for whom prevalence may be higher than 50%.

The development of lower and upper limits of RIs and RCs has inherent limitations due to its distribution-based approach. Thresholds (also known as clinical decision limits) are established based on clinically important outcomes.^3^ Using data from the same cohort, investigators (including P.C.P.) have previously identified a ferritin threshold of 17 to 23.7 ng/mL for iron deficiency in children aged 1 to 3 years using a physiologic outcome (hemoglobin) and a functional outcome (cognition).^15,57^ However, iron deficiency thresholds do not provide an upper limit, which also has clinical utility. Furthermore, the iron deficiency threshold may vary across age, as seen in the continuous lower limit of the curves, suggesting a single pediatric threshold may not suffice.

Our interactive web-based tool is intended to complement rather than replace existing current laboratory reporting standards, which include reporting RIs. In addition, the tool contextualizes iron deficiency thresholds in terms of normative and optimal levels that vary across age and sex. However, the tool requires further validation by clinical and laboratory users. Future work will focus on expanding the underlying model using international datasets to improve generalizability.

### Strengths and Limitations

Strengths of our study include the large sample size of preadolescent children, including those in the higher-risk age group of 1 to 3 years, use of data from a healthy community cohort, application of prespecified optimality criteria, use of rigorous statistical methods following Clinical Laboratory Standards Institute guidelines, use of multiple imputation to address missing data on optimality criteria, and contextualizing our findings alongside the WHO and recently proposed ASH iron deficiency thresholds. However, several limitations should be considered. Data were collected from a single urban center in Canada with exclusion of non–English-speaking families, which may limit generalizability. Therefore, the curves and intervals should not be adopted internationally without local verification. It is notable that physiologically based thresholds for young children from our Canadian cohort^15^ are similar to those from US^17,18,19^ and 12 other countries.^20^ Excluding children with a CRP level greater than 0.5 mg/dL may have excluded some children with low-grade inflammation related to adiposity. Previous research in this cohort that included several of our investigators^33^ examined the complex association among ferritin levels, BMI *z* scores, and CRP levels in young children and found no interaction between BMI *z* score and CRP level. In addition, we lacked sufficient representation of neonates younger than 2 weeks and children older than 10 years. Iron disorders are rare in neonates, but iron deficiency is prevalent in adolescent females. Physiologically based iron deficiency thresholds have been reported in a cohort of nonpregnant women, including those as young as 15 years.^17,19^ Missingness was assumed to be missing at random; however, this assumption cannot be empirically confirmed from observed data alone, which remains an inherent limitation.

## Conclusions

In this cross-sectional study, we derived ferritin RCs and OCs from a low-risk population of preadolescent children. In contrast to RIs, the curve approach addressed the limitations of age partitioning and sample size. However, the lower limit of curves and intervals were substantially lower than physiologically based thresholds to define iron deficiency. These findings further inform the current discussion regarding the distributional vs physiologic approaches to interpreting ferritin measurements and guiding clinical decision-making.

## Notes

Supplement 1. **eTable 1.** Number of Children Used in Estimations of Reference and Optimal Ferritin Curves by Sex and Standardized Age Groups **eTable 2.** Age- and Sex-Specific Reference Intervals (RIs) and Optimal Intervals (OIs) for Children Aged 0 to 131 Months **eFigure 1.** Ferritin Optimal Curves for Males Aged 2 Weeks to 10 Years Estimated Using Multiple Imputation First Then Delete (MID) and Delete First Then Multiple Imputation (DMI) **eFigure 2.** Ferritin Optimal Curves for Females Aged 2 Weeks to 10 Years Estimated Using Multiple Imputation First Then Delete (MID) and Delete First Then Multiple Imputation (DMI) **eTable 3.** Characteristics of Study Participants in Imputed and Complete Case Datasets Used for Optimal Curve Estimation Prior to Excluding Those Not Meeting Optimality Criteria **eFigure 3.** Ferritin Optimal Curves for Males Aged 2 Weeks to 10 Years Comparing Estimates Obtained Using the Multiple Imputation Then Delete (MID) Approach and Complete Case Analysis **eFigure 4.** Ferritin Optimal Curves for Females Aged 2 Weeks to 10 Years Comparing Estimates Obtained Using the Multiple Imputation Then Delete (MID) Approach and Complete Case Analysis

Supplement 2.
Data Sharing Statement
