# Supplementary material for: Ferritin Reference Curves and Optimal Curves in Preadolescent Children
Source: JAMA Netw Open. 2026 May 15;9(5):e2613041. doi: 10.1001/jamanetworkopen.2026.13041 (PMC13179555; doi:10.1001/jamanetworkopen.2026.13041)
Supplement: Supplement 1. — eTable 1. Number of Children Used in Estimations of Reference and Optimal Ferritin Curves by Sex and Standardized Age Groups eTable 2. Age- and Sex-Specific Reference Intervals (RIs) and Optimal Intervals (OIs) for Children Aged 0 to 131 Months eFigure 1. Ferritin Optimal Curves for Males Aged 2 Weeks to 10 Years Estimated Using Multiple Imputation First Then Delete (MID) and Delete First Then Multiple Imputation (DMI) eFigure 2. Ferritin Optimal Curves for Females Aged 2 Weeks to 10 Years Estimated Using Multiple Imputation First Then Delete (MID) and Delete First Then Multiple Imputation (DMI) eTable 3. Characteristics of Study Participants in Imputed and Complete Case Datasets Used for Optimal Curve Estimation Prior to Excluding Those Not Meeting Optimality Criteria eFigure 3. Ferritin Optimal Curves for Males Aged 2 Weeks to 10 Years Comparing Estimates Obtained Using the Multiple Imputation Then Delete (MID) Approach and Complete Case Analysis eFigure 4. Ferritin Optimal Curves for Females Aged 2 Weeks to 10 Years Comparing Estimates Obtained Using the Multiple Imputation Then Delete (MID) Approach and Complete Case Analysis [file jamanetwopen-e2613041-s001.pdf]

Supplementary Online Content

Bijelić V, Momoli F, Liebman M, et al. Ferritin reference curves and optimal curves in preadolescent children. *JAMA Netw Open.* 2026;9(5):e2613041. doi:10.1001/jamanetworkopen.2026.13041

eTable 1. Number of Children Used in Estimations of Reference and Optimal Ferritin Curves by Sex and Standardized Age Groups

eTable 2. Age- and Sex-Specific Reference Intervals (RIs) and Optimal Intervals (OIs) for Children Aged 0 to 131 Months

eFigure 1. Ferritin Optimal Curves for Males Aged 2 Weeks to 10 Years Estimated Using Multiple Imputation First Then Delete (MID) and Delete First Then Multiple Imputation (DMI)

eFigure 2. Ferritin Optimal Curves for Females Aged 2 Weeks to 10 Years Estimated Using Multiple Imputation First Then Delete (MID) and Delete First Then Multiple Imputation (DMI)

eTable 3. Characteristics of Study Participants in Imputed and Complete Case Datasets Used for Optimal Curve Estimation Prior to Excluding Those Not Meeting Optimality Criteria

eFigure 3. Ferritin Optimal Curves for Males Aged 2 Weeks to 10 Years Comparing Estimates Obtained Using the Multiple Imputation Then Delete (MID) Approach and Complete Case Analysis

eFigure 4. Ferritin Optimal Curves for Females Aged 2 Weeks to 10 Years Comparing Estimates Obtained Using the Multiple Imputation Then Delete (MID) Approach and Complete Case Analysis

This supplementary material has been provided by the authors to give readers additional information about their work.

© 2026 Bijelić V et al. *JAMA Network Open*.

eTable 1. Number of Children Used in Estimations of Reference and Optimal Ferritin Curves by Sex and Standardized Age Groups

Characteristic	Reference Curves		Optimal Curves	
	**Female N = 2,322**	**Male N = 2,613**	**Female N = 1,721**	**Male N = 1,909**
Age Group	n (%)	n (%)	n (%)	n (%)
2 weeks - 2 months	12 (0.5%)	22 (0.8%)	10 (0.6%)	13 (0.7%)
3 - 5 months	25 (1.1%)	25 (1.0%)	14 (0.8%)	13 (0.7%)
6 - 7 months	68 (2.9%)	65 (2.5%)	47 (2.7%)	41 (2.1%)
9 - 11 months	113 (4.9%)	145 (5.5%)	73 (4.2%)	80 (4.2%)
12 - 14 months	165 (7.1%)	185 (7.1%)	104 (6.0%)	107 (5.6%)
15 - 17 months	120 (5.2%)	140 (5.4%)	76 (4.4%)	98 (5.1%)
18 - 20 months	161 (6.9%)	165 (6.3%)	111 (6.4%)	106 (5.6%)
21 - 23 months	45 (1.9%)	62 (2.4%)	29 (1.7%)	36 (1.9%)
24 - 26 months	214 (9.2%)	208 (8.0%)	154 (8.9%)	163 (8.5%)
27 - 29 months	49 (2.1%)	53 (2.0%)	35 (2.0%)	41 (2.1%)
30 - 32 months	29 (1.2%)	43 (1.6%)	18 (1.0%)	21 (1.1%)
33 - 35 months	28 (1.2%)	44 (1.7%)	21 (1.2%)	30 (1.6%)
3 - <4 years	326 (14%)	319 (12%)	261 (15%)	252 (13%)
4 - <5 years	288 (12%)	354 (14%)	232 (13%)	288 (15%)
5 - <6 years	249 (11%)	266 (10%)	176 (10%)	203 (11%)
6 - <7 years	110 (4.7%)	146 (5.6%)	92 (5.3%)	117 (6.1%)
7 - <8 years	110 (4.7%)	102 (3.9%)	94 (5.5%)	83 (4.3%)
8 - <9 years	101 (4.3%)	112 (4.3%)	88 (5.1%)	90 (4.7%)
9 - <10 years	65 (2.8%)	89 (3.4%)	50 (2.9%)	69 (3.6%)
10 - <11 years	44 (1.9%)	68 (2.6%)	36 (2.1%)	58 (3.0%)

© 2026 Bijelić V et al. *JAMA Network Open*.

eTable 2. Age- and Sex-Specific Reference Intervals (RIs) and Optimal Intervals (OIs) for Children Aged 0 to 131 Months

Month	Lower Limit		Upper Limit	
	Optimal Limit	Reference Limit	Optimal Limit	Reference Limit
**Males**				
0	31.3 (15.7-46.9)	30.6 (13.9-53.1)	620.8 (149.5-1092.1)	790.6 (466.6-1219.6)
1	25.9 (14.7-37.1)	23.2 (12.2-35.8)	484.3 (283.6-685.1)	570.7 (382.6-764.1)
2	21.5 (13.4-29.6)	17.5 (10.6-25)	383 (260.3-505.6)	414.8 (317.9-524.3)
3	18 (12.2-23.9)	13.6 (9.3-17.9)	306.4 (226.4-386.5)	309.3 (253.7-376.5)
4	15.3 (11-19.5)	10.8 (8-13.9)	248.3 (194-302.6)	237.5 (199.9-288)
5	13.1 (9.9-16.3)	8.9 (7-11.4)	204 (165.7-242.3)	190.3 (156.7-230.8)
6	11.4 (8.9-13.8)	7.7 (6.3-9.7)	170.2 (142.1-198.3)	155.8 (128.4-191.7)
7	10 (8.1-11.9)	6.9 (5.7-8.5)	144.4 (123-165.8)	133.3 (110.5-161.9)
8	9 (7.5-10.5)	6.4 (5.4-7.7)	124.4 (107.6-141.3)	118 (100.2-139)
9	8.2 (6.9-9.4)	6 (5.1-7.1)	108.9 (95.3-122.5)	106.7 (94.5-123.4)
10	7.5 (6.4-8.6)	5.8 (4.9-7)	96.7 (85.5-107.9)	98.3 (88.6-112.1)
11	7 (6.1-7.9)	5.7 (4.8-6.8)	87.1 (77.6-96.5)	92 (83.4-103.3)
12	6.6 (5.8-7.4)	5.5 (4.8-6.6)	79.4 (71.2-87.6)	86.3 (78-95.8)
13	6.3 (5.6-7.1)	5.4 (4.7-6.5)	73.4 (66.2-80.6)	81.3 (73.8-90.9)
14	6.1 (5.4-6.8)	5.3 (4.6-6.2)	68.6 (62.2-75.1)	76.4 (69.9-85.2)
15	6 (5.3-6.6)	5.2 (4.6-6)	64.9 (59-70.8)	72 (66.1-79.7)
16	5.9 (5.2-6.5)	5.1 (4.6-5.8)	62.1 (56.7-67.6)	68.2 (63-74.7)
17	5.8 (5.2-6.5)	5.1 (4.5-5.7)	60.1 (54.9-65.3)	65.2 (60.7-70.7)
18	5.9 (5.3-6.5)	5 (4.6-5.7)	58.7 (53.7-63.7)	62.6 (57.9-68.5)
19	6 (5.3-6.6)	5.1 (4.6-5.7)	58 (53.1-62.8)	61.1 (56.1-67)
20	6.1 (5.5-6.7)	5.2 (4.7-5.9)	57.7 (53-62.5)	60.5 (55.6-66.1)
21	6.3 (5.7-6.9)	5.4 (4.8-6)	57.9 (53.3-62.6)	60.4 (55.8-65.6)
22	6.5 (5.9-7.1)	5.6 (5.1-6.2)	58.4 (53.8-63.1)	61 (56.9-66.3)
23	6.7 (6.1-7.4)	5.8 (5.2-6.5)	59.2 (54.5-63.8)	61.8 (58-67.1)
24	7 (6.3-7.7)	6.1 (5.5-6.8)	60 (55.1-64.8)	62.6 (58.5-68)
25	7.2 (6.5-8)	6.3 (5.6-7.1)	60.7 (55.7-65.8)	63.4 (59-69.7)
26	7.5 (6.7-8.2)	6.5 (5.8-7.3)	61.4 (56.2-66.5)	63.9 (59.1-70.4)
27	7.7 (6.9-8.4)	6.7 (6-7.4)	61.7 (56.4-67)	64.1 (58.9-70.4)

© 2026 Bijelić V et al. *JAMA Network Open*.

28	7.9 (7.1-8.6)	6.9 (6.2-7.5)	61.9 (56.5-67.2)	64.1 (58.9-70.3)
29	8 (7.2-8.8)	7 (6.3-7.7)	61.9 (56.5-67.2)	63.6 (58.1-70.3)
30	8.2 (7.4-9)	7.2 (6.4-7.9)	61.8 (56.4-67.1)	63.1 (57.3-69.5)
31	8.3 (7.5-9.1)	7.3 (6.5-8.1)	61.6 (56.2-67)	62.9 (56.9-69.2)
32	8.4 (7.6-9.2)	7.4 (6.6-8.4)	61.4 (56.1-66.8)	62.7 (56.6-69.4)
33	8.5 (7.7-9.4)	7.5 (6.7-8.5)	61.3 (56.1-66.5)	62.5 (56.7-69.6)
34	8.7 (7.9-9.5)	7.6 (6.8-8.6)	61.3 (56.3-66.3)	62.4 (57.1-68.7)
35	8.8 (8-9.6)	7.8 (7.1-8.8)	61.4 (56.6-66.2)	62.4 (57.6-68.5)
36	9 (8.2-9.8)	8 (7.2-9)	61.6 (57-66.3)	62.9 (58.2-68)
37	9.2 (8.4-10)	8.2 (7.4-9.1)	62 (57.4-66.6)	63.3 (58.4-68)
38	9.4 (8.6-10.2)	8.4 (7.6-9.4)	62.4 (57.8-67)	63.8 (58.5-69.2)
39	9.6 (8.8-10.4)	8.7 (7.8-9.7)	63 (58.3-67.7)	64.5 (58.9-70.2)
40	9.8 (9-10.6)	8.9 (8-9.9)	63.7 (58.9-68.5)	65.2 (59.3-71.4)
41	10 (9.2-10.9)	9.2 (8.3-10.2)	64.5 (59.6-69.4)	66 (60.4-72.4)
42	10.3 (9.5-11.1)	9.4 (8.5-10.4)	65.4 (60.4-70.4)	66.7 (61.5-73.5)
43	10.5 (9.7-11.4)	9.6 (8.7-10.6)	66.3 (61.1-71.4)	67.5 (62.3-74.3)
44	10.8 (9.9-11.6)	9.8 (8.9-10.9)	67.1 (61.8-72.3)	68.1 (62.8-75.6)
45	11 (10.1-11.8)	10 (9-11.1)	67.8 (62.5-73.1)	68.4 (62.9-76)
46	11.2 (10.3-12)	10.1 (9.2-11.2)	68.4 (63.1-73.6)	68.6 (63.1-77)
47	11.3 (10.5-12.1)	10.2 (9.3-11.3)	68.7 (63.7-73.8)	68.4 (63.5-76.6)
48	11.4 (10.6-12.2)	10.3 (9.4-11.3)	69 (64.1-73.8)	68.3 (63.5-75.6)
49	11.6 (10.8-12.3)	10.4 (9.3-11.4)	69 (64.3-73.8)	68.1 (63.2-74.8)
50	11.6 (10.8-12.5)	10.4 (9.3-11.5)	69.1 (64.4-73.7)	68 (62.7-74.3)
51	11.7 (10.9-12.6)	10.5 (9.5-11.6)	69.1 (64.4-73.7)	68 (62.5-74.2)
52	11.8 (10.9-12.7)	10.6 (9.5-11.8)	69.1 (64.3-73.8)	67.8 (62.5-74)
53	11.9 (11-12.8)	10.7 (9.7-11.9)	69.1 (64.3-73.9)	67.9 (63-74.6)
54	12 (11.1-12.9)	10.8 (9.8-12)	69.2 (64.5-73.9)	68.3 (63.3-74.1)
55	12.1 (11.2-13)	11 (10-12.1)	69.3 (64.7-74)	68.9 (63.8-74.8)
56	12.2 (11.3-13.1)	11.2 (10.2-12.2)	69.5 (64.9-74.1)	69.6 (64-75.5)
57	12.3 (11.4-13.2)	11.3 (10.4-12.3)	69.6 (65-74.1)	70.4 (65.1-76.1)
58	12.3 (11.4-13.3)	11.5 (10.6-12.5)	69.6 (65.1-74.2)	71 (65.8-76.5)
59	12.4 (11.5-13.3)	11.6 (10.7-12.6)	69.6 (65.1-74.1)	71.4 (65.9-76.9)

© 2026 Bijelić V et al. *JAMA Network Open*.

60	12.5 (11.6-13.4)	11.7 (10.9-12.7)	69.5 (65.1-73.9)	71.5 (65.9-76.9)
61	12.5 (11.6-13.4)	11.8 (10.9-12.8)	69.3 (64.9-73.7)	71.5 (66.2-76.8)
62	12.5 (11.6-13.5)	11.8 (10.9-12.9)	69.1 (64.6-73.5)	71.4 (66.6-77)
63	12.5 (11.6-13.5)	11.9 (10.9-13)	68.8 (64.3-73.3)	71.3 (66.2-76.9)
64	12.6 (11.6-13.5)	11.9 (10.8-13.1)	68.5 (63.8-73.2)	71.2 (65.6-76.5)
65	12.6 (11.5-13.6)	11.9 (10.8-13.2)	68.2 (63.4-73.1)	71 (65.4-76.5)
66	12.6 (11.5-13.6)	12 (10.9-13.3)	68 (63-73)	71.1 (65.5-76.7)
67	12.6 (11.5-13.7)	12.1 (10.9-13.4)	67.8 (62.8-72.8)	71 (65.5-76.7)
68	12.7 (11.6-13.8)	12.1 (11-13.5)	67.7 (62.7-72.7)	71 (65.9-76.7)
69	12.7 (11.6-13.8)	12.2 (11.1-13.5)	67.6 (62.6-72.7)	71.3 (65.6-76.6)
70	12.8 (11.6-13.9)	12.3 (11.1-13.5)	67.7 (62.5-72.8)	71.4 (65.3-76.9)
71	12.8 (11.7-14)	12.3 (11.2-13.5)	67.7 (62.5-73)	71.4 (65.7-77.6)
72	12.9 (11.7-14.1)	12.3 (11.2-13.6)	67.9 (62.6-73.3)	71.3 (65.3-78.4)
73	13 (11.8-14.2)	12.3 (11.1-13.6)	68.1 (62.7-73.6)	70.8 (65.1-78)
74	13.1 (11.9-14.4)	12.3 (11.1-13.5)	68.4 (62.9-73.9)	70.4 (64.9-78.2)
75	13.3 (12-14.5)	12.4 (11-13.6)	68.8 (63.2-74.3)	69.9 (64.8-77.5)
76	13.4 (12.2-14.7)	12.3 (11-13.6)	69.2 (63.6-74.9)	69.4 (63.6-77.5)
77	13.6 (12.3-14.8)	12.3 (11-13.7)	69.7 (64-75.5)	69.3 (63.2-78.3)
78	13.7 (12.4-15)	12.4 (10.9-13.9)	70.3 (64.5-76.2)	69.3 (62.4-78.5)
79	13.9 (12.6-15.2)	12.5 (11-14.1)	71 (65.1-76.9)	69.3 (62.3-78.6)
80	14.1 (12.8-15.4)	12.7 (11.1-14.2)	71.8 (65.9-77.7)	70 (62.9-78.5)
81	14.3 (13-15.7)	12.9 (11.4-14.3)	72.6 (66.7-78.4)	70.9 (64.1-78.3)
82	14.5 (13.2-15.9)	13.1 (11.7-14.6)	73.4 (67.6-79.2)	72.1 (65.3-79)
83	14.7 (13.3-16.1)	13.4 (12-15)	74.2 (68.4-80)	73.5 (67.3-80.4)
84	14.9 (13.5-16.3)	13.7 (12.3-15.3)	75 (69.1-80.8)	74.7 (68.7-81.9)
85	15.1 (13.6-16.5)	14 (12.4-15.6)	75.7 (69.7-81.7)	75.8 (69.4-82.6)
86	15.2 (13.7-16.7)	14.2 (12.6-15.8)	76.3 (70.1-82.4)	77 (70.4-84.3)
87	15.3 (13.8-16.8)	14.3 (12.9-16)	76.7 (70.4-83)	77.4 (71-85.2)
88	15.4 (13.9-16.9)	14.5 (13-16.1)	77.1 (70.6-83.6)	78.2 (71.1-86.4)
89	15.4 (13.9-16.9)	14.6 (13.2-16.3)	77.3 (70.6-84)	78.5 (70.9-87.5)
90	15.4 (14-16.9)	14.7 (13.2-16.5)	77.4 (70.4-84.4)	78.6 (70.4-88.8)
91	15.5 (14-16.9)	14.8 (13.2-16.5)	77.5 (70.2-84.7)	78.6 (69.9-89.2)

© 2026 Bijelić V et al. *JAMA Network Open*.

92	15.5 (14-16.9)	14.8 (13.2-16.8)	77.4 (70-84.9)	78.7 (69.4-90.9)
93	15.4 (14-16.9)	14.8 (13.2-16.9)	77.4 (69.8-84.9)	78.7 (69.3-90.2)
94	15.4 (14-16.9)	14.8 (13.2-16.8)	77.3 (69.7-84.8)	78.6 (68.7-90.6)
95	15.4 (14-16.8)	14.8 (13.2-16.8)	77.2 (69.6-84.7)	78.5 (68.8-90.1)
96	15.4 (14-16.8)	14.8 (13.1-16.8)	77.1 (69.6-84.6)	78 (69.4-89.3)
97	15.4 (14-16.8)	14.8 (13-16.7)	77.1 (69.6-84.6)	77.7 (69.1-88.6)
98	15.4 (14-16.8)	14.8 (13.1-16.5)	77.1 (69.7-84.6)	77.5 (69.1-88.2)
99	15.4 (14-16.9)	14.9 (13.1-16.5)	77.3 (69.9-84.6)	77.4 (69.9-87.7)
100	15.5 (14-17)	15 (13.1-16.5)	77.5 (70.3-84.8)	77.5 (70.1-87.8)
101	15.6 (14.1-17.1)	15 (13.2-16.5)	77.9 (70.7-85.1)	77.7 (70.3-87.5)
102	15.7 (14.1-17.3)	15.2 (13.4-16.6)	78.4 (71.3-85.6)	78.2 (70.6-87.1)
103	15.8 (14.2-17.5)	15.3 (13.3-17)	79 (71.8-86.3)	78.6 (71.2-87.5)
104	16 (14.3-17.8)	15.5 (13.2-17.3)	79.8 (72.5-87.1)	79.2 (71-87.1)
105	16.2 (14.4-18)	15.7 (13.2-17.7)	80.6 (73.2-88.1)	80 (71.5-87.8)
106	16.4 (14.5-18.3)	15.8 (13.5-17.9)	81.6 (74-89.1)	81 (72-89.3)
107	16.7 (14.7-18.6)	16.1 (13.8-18.1)	82.6 (75-90.2)	82 (73.1-90.6)
108	16.9 (15-18.9)	16.4 (14.1-18.4)	83.7 (76-91.3)	82.8 (74.4-92)
109	17.2 (15.2-19.1)	16.6 (14.5-19)	84.7 (77-92.5)	84.3 (76-93)
110	17.4 (15.4-19.4)	16.8 (14.6-19.6)	85.8 (77.8-93.7)	85.6 (77.5-94.1)
111	17.7 (15.6-19.7)	17.1 (14.7-20.4)	86.7 (78.5-94.9)	87 (78.2-95.5)
112	17.9 (15.8-19.9)	17.4 (14.9-20.8)	87.5 (79-96)	87.9 (78.8-97.4)
113	18.1 (16-20.1)	17.6 (14.9-20.8)	88.1 (79.3-96.9)	88.4 (78.3-98.9)
114	18.2 (16.2-20.2)	17.7 (15.2-20.5)	88.4 (79.3-97.5)	88.7 (77.9-99.5)
115	18.3 (16.3-20.3)	17.8 (15.3-20.5)	88.5 (79.2-97.9)	88.3 (78.1-98.9)
116	18.3 (16.3-20.3)	17.8 (15.5-20.6)	88.4 (78.7-98)	88.2 (77.1-99)
117	18.3 (16.3-20.3)	17.8 (15.6-20.5)	87.9 (78-97.9)	87.9 (75.7-98.8)
118	18.2 (16.3-20.2)	17.7 (15.5-20.3)	87.2 (77.1-97.4)	87.3 (74.2-98)
119	18.1 (16.1-20)	17.5 (15.5-20)	86.2 (75.9-96.6)	86 (73.4-97.3)
120	17.9 (15.9-19.9)	17.4 (15.4-19.7)	85 (74.5-95.5)	84.9 (71.8-96.5)
121	17.7 (15.7-19.7)	17.2 (15.3-19.5)	83.5 (72.9-94.1)	83.6 (70.4-96.4)
122	17.4 (15.4-19.4)	17.1 (14.9-19.5)	81.9 (71.3-92.5)	82.4 (68.4-95)
123	17.1 (15.1-19.2)	16.9 (14.6-19.4)	80.2 (69.6-90.9)	81.3 (67.2-94.4)

© 2026 Bijelić V et al. *JAMA Network Open*.

124	16.9 (14.7-19)	16.7 (14.3-19.6)	78.6 (67.9-89.3)	80.2 (65.5-93.5)
125	16.6 (14.3-18.9)	16.5 (14.1-19.5)	76.9 (66.2-87.7)	78.8 (63.9-92.5)
126	16.4 (14-18.7)	16.3 (13.8-19.3)	75.4 (64.6-86.3)	77.7 (62.5-91.1)
127	16.1 (13.6-18.7)	16.1 (13.6-19.1)	74.1 (63.1-85)	76.8 (62-89.6)
128	16 (13.3-18.7)	16 (13.3-19.2)	72.8 (61.6-84.1)	75.5 (61.2-89.3)
129	15.8 (12.9-18.7)	16 (13.1-19.2)	71.7 (59.9-83.5)	74.6 (60.2-88.3)
130	15.7 (12.5-18.9)	16.1 (12.7-19.3)	70.7 (58.1-83.4)	74.1 (58.8-89.3)
131	15.6 (12-19.2)	16.2 (12.2-19.9)	69.9 (56.1-83.6)	73.9 (55.9-90.9)
**Females**				
0	38 (6.5-69.4)	25.4 (14.1-54.5)	841 (424.6-1257.5)	733.9 (438.5-1251.7)
1	29.6 (8.7-50.4)	21.3 (12.9-39.3)	635.2 (380.5-889.9)	577.4 (379.7-892.1)
2	23.3 (9.5-37.2)	17.8 (11.7-29.1)	485 (329.7-640.4)	456.3 (328.1-641.2)
3	18.7 (9.4-28)	14.9 (10.4-22.1)	376 (280.1-472)	368.1 (278.2-475.6)
4	15.4 (9-21.7)	12.7 (9.4-17.4)	296.6 (235-358.3)	300.4 (238.4-372)
5	12.9 (8.5-17.2)	11 (8.4-13.9)	238.6 (195.9-281.3)	248.4 (202.9-292.9)
6	11 (7.9-14.2)	9.5 (7.6-11.7)	196.2 (163.9-228.5)	208.3 (174.4-243.6)
7	9.7 (7.4-12)	8.5 (7-10.1)	164.9 (138.8-190.9)	178.3 (152.1-205.6)
8	8.7 (6.9-10.4)	7.6 (6.4-9)	141.5 (119.7-163.2)	154.7 (132.9-174.7)
9	8 (6.6-9.4)	7 (5.9-8.1)	123.8 (105.4-142.2)	136.2 (118.1-152.8)
10	7.4 (6.3-8.6)	6.5 (5.6-7.5)	110.3 (94.6-126)	121.3 (106.8-137.2)
11	7 (6.1-8)	6.2 (5.3-7.1)	99.9 (86.4-113.4)	110.5 (97.9-124.7)
12	6.8 (5.9-7.7)	5.9 (5.1-6.8)	92 (80.2-103.7)	101.7 (91.1-114.5)
13	6.6 (5.8-7.4)	5.7 (4.9-6.5)	85.8 (75.5-96.1)	95.1 (85.4-105.6)
14	6.5 (5.8-7.3)	5.6 (4.9-6.4)	81.1 (72-90.3)	89.2 (81.2-98.5)
15	6.5 (5.8-7.3)	5.5 (4.8-6.4)	77.6 (69.3-85.8)	84.8 (77.8-93.4)
16	6.6 (5.9-7.3)	5.5 (4.9-6.3)	75 (67.3-82.6)	81.4 (74.8-89.5)
17	6.7 (6-7.4)	5.6 (4.9-6.3)	73.1 (65.9-80.3)	78.9 (71.8-86.2)
18	6.8 (6.1-7.5)	5.6 (5-6.3)	71.8 (64.9-78.7)	77.2 (70-83.8)
19	7 (6.3-7.7)	5.7 (5.1-6.4)	71 (64.2-77.8)	75.7 (68.6-81.9)
20	7.2 (6.5-8)	5.9 (5.2-6.5)	70.6 (63.9-77.2)	74.7 (67.9-80.6)
21	7.5 (6.7-8.2)	6.1 (5.3-6.7)	70.4 (63.8-76.9)	74 (67.5-79.9)
22	7.7 (7-8.5)	6.3 (5.5-7)	70.3 (63.8-76.9)	73.6 (67.2-79.5)

© 2026 Bijelić V et al. *JAMA Network Open*.

23	8 (7.2-8.8)	6.5 (5.6-7.2)	70.4 (63.8-77)	73.1 (66.9-79.6)
24	8.2 (7.4-9.1)	6.7 (5.8-7.5)	70.3 (63.7-77)	72.8 (66.5-79.4)
25	8.5 (7.6-9.3)	6.9 (5.9-7.7)	70.2 (63.4-77)	72.3 (65.9-79)
26	8.7 (7.7-9.6)	7 (6.1-7.9)	69.8 (62.9-76.6)	71.7 (65.1-78.4)
27	8.8 (7.9-9.7)	7.2 (6.3-8.1)	69.1 (62.2-75.9)	70.7 (64-77.9)
28	8.9 (7.9-9.8)	7.4 (6.5-8.3)	68.2 (61.4-75)	70 (62.8-77.2)
29	9 (8-9.9)	7.5 (6.6-8.5)	67.2 (60.5-74)	69.4 (61.8-76.5)
30	9 (8-10)	7.6 (6.7-8.6)	66.2 (59.5-72.9)	68.8 (61-75.7)
31	9 (8-10.1)	7.7 (6.9-8.7)	65.3 (58.7-71.9)	68.2 (60.3-75.1)
32	9.1 (8.1-10.1)	7.9 (7-8.9)	64.5 (58.1-70.9)	67.5 (59.3-74.4)
33	9.1 (8.1-10.2)	8 (7.1-9)	63.9 (57.8-70.1)	67.1 (59.1-73.7)
34	9.2 (8.2-10.2)	8.2 (7.3-9.1)	63.6 (57.7-69.4)	66.8 (59.4-73.2)
35	9.3 (8.4-10.3)	8.4 (7.5-9.2)	63.4 (57.9-69)	66.8 (60-73)
36	9.4 (8.5-10.4)	8.6 (7.7-9.5)	63.5 (58.1-68.8)	66.8 (60.7-72.8)
37	9.6 (8.6-10.5)	8.8 (7.9-9.8)	63.7 (58.4-69)	66.9 (61.1-73)
38	9.7 (8.8-10.7)	9 (8-10.1)	64 (58.6-69.5)	67.3 (61.3-73.3)
39	9.9 (8.9-10.9)	9.2 (8.1-10.3)	64.6 (59-70.1)	67.7 (61.6-73.6)
40	10.1 (9.1-11.1)	9.4 (8.3-10.5)	65.2 (59.5-70.9)	68.4 (61.9-73.9)
41	10.3 (9.3-11.3)	9.6 (8.5-10.6)	65.9 (60.1-71.7)	69.1 (62.4-74.7)
42	10.5 (9.5-11.5)	9.8 (8.7-10.8)	66.7 (60.8-72.6)	69.8 (63-75.6)
43	10.7 (9.7-11.7)	10 (8.8-11.1)	67.5 (61.6-73.5)	70.4 (63.7-76.5)
44	10.9 (9.9-12)	10.3 (9-11.3)	68.4 (62.3-74.4)	71 (64.5-77.6)
45	11.1 (10.1-12.2)	10.4 (9.2-11.4)	69.2 (63.2-75.2)	71.8 (65.5-78.5)
46	11.3 (10.3-12.3)	10.5 (9.4-11.6)	70 (64-75.9)	72.6 (66.1-79.5)
47	11.5 (10.5-12.5)	10.7 (9.6-11.8)	70.7 (64.9-76.5)	73.5 (66.6-80.1)
48	11.6 (10.6-12.6)	10.8 (9.8-11.9)	71.4 (65.7-77.1)	74.2 (67.7-80.6)
49	11.8 (10.8-12.8)	10.9 (9.8-12.1)	72 (66.3-77.7)	74.8 (68.4-81.7)
50	11.9 (10.9-12.9)	11 (9.9-12.2)	72.6 (66.8-78.3)	75.6 (68.9-82.2)
51	12 (10.9-13.1)	11.1 (9.9-12.2)	73.1 (67.2-78.9)	76.1 (69.7-82.9)
52	12.1 (11-13.2)	11.2 (10-12.3)	73.6 (67.6-79.5)	76.8 (70.4-83.6)
53	12.1 (11-13.3)	11.2 (10.1-12.3)	74 (67.9-80)	77.3 (70.6-84.2)
54	12.2 (11-13.4)	11.3 (10.1-12.5)	74.3 (68.2-80.4)	77.8 (71.1-85.1)

© 2026 Bijelić V et al. *JAMA Network Open*.

55	12.2 (11-13.4)	11.4 (10.1-12.6)	74.6 (68.5-80.7)	78.3 (71.5-85.5)
56	12.3 (11-13.5)	11.4 (10.1-12.6)	74.9 (68.8-81.1)	78.9 (72.2-86.1)
57	12.3 (11-13.6)	11.4 (10.1-12.6)	75.2 (68.9-81.4)	79.1 (73-86.9)
58	12.3 (11-13.6)	11.4 (10.1-12.7)	75.4 (69.1-81.6)	79.4 (73.8-87.9)
59	12.4 (11-13.7)	11.4 (10-12.7)	75.6 (69.3-81.9)	79.9 (73.8-88.6)
60	12.4 (11.1-13.7)	11.5 (10.1-12.7)	75.7 (69.4-82.1)	80.1 (73.7-89.7)
61	12.5 (11.2-13.8)	11.5 (10.2-12.7)	75.9 (69.4-82.3)	80.2 (73.6-90.1)
62	12.5 (11.3-13.8)	11.6 (10.3-12.8)	76 (69.3-82.7)	80.7 (73.3-90.3)
63	12.6 (11.3-13.9)	11.6 (10.3-13)	76 (69-83.1)	80.6 (73-90.3)
64	12.7 (11.4-14)	11.7 (10.3-13.1)	76 (68.6-83.5)	80.7 (72.7-90.7)
65	12.8 (11.5-14.1)	11.8 (10.4-13.2)	75.9 (68.1-83.7)	80.7 (72.1-91.1)
66	12.9 (11.5-14.2)	11.9 (10.5-13.2)	75.7 (67.7-83.6)	80.5 (72.3-91)
67	12.9 (11.6-14.3)	11.9 (10.6-13.2)	75.4 (67.5-83.2)	80.5 (72.2-90.6)
68	13 (11.7-14.3)	12 (10.6-13.3)	75 (67.3-82.7)	80.1 (72.5-89.8)
69	13.1 (11.8-14.4)	12.1 (10.6-13.3)	74.6 (67.2-82)	79.7 (72.6-89.2)
70	13.2 (11.8-14.6)	12.1 (10.6-13.5)	74.2 (67.1-81.3)	79 (72.1-88.7)
71	13.3 (11.9-14.7)	12.2 (10.7-13.6)	73.8 (66.9-80.6)	78.3 (71.5-87.9)
72	13.4 (11.9-14.9)	12.2 (10.7-13.7)	73.4 (66.8-80)	77.5 (71.3-86.8)
73	13.5 (12-15.1)	12.3 (10.8-13.8)	73.1 (66.6-79.6)	76.6 (69.9-85.6)
74	13.7 (12-15.3)	12.3 (10.8-14)	72.8 (66.4-79.3)	75.7 (69-84.1)
75	13.8 (12.1-15.5)	12.3 (10.8-14.2)	72.6 (66.1-79.1)	75 (68.2-82.5)
76	14 (12.2-15.7)	12.3 (10.8-14.5)	72.5 (65.8-79.1)	74.1 (66.2-81.2)
77	14.1 (12.3-15.9)	12.3 (10.9-14.7)	72.4 (65.5-79.2)	73 (64.6-80.4)
78	14.2 (12.3-16.1)	12.3 (11-14.8)	72.3 (65.2-79.3)	72.4 (63.4-80.4)
79	14.4 (12.4-16.3)	12.4 (10.9-15)	72.3 (65-79.5)	72 (62.7-80.4)
80	14.5 (12.5-16.4)	12.4 (11-15.2)	72.3 (64.8-79.8)	71.8 (62.2-80.9)
81	14.6 (12.7-16.6)	12.6 (10.9-15.3)	72.4 (64.7-80.1)	71.8 (62.2-80.5)
82	14.7 (12.8-16.7)	12.7 (11.1-15.6)	72.6 (64.7-80.6)	72.4 (62.2-81.1)
83	14.9 (12.9-16.8)	12.9 (11.1-15.7)	73 (64.9-81.2)	72.9 (62.6-82.1)
84	15 (13.1-17)	13.1 (11.2-15.8)	73.6 (65.1-82)	73.9 (62.9-82.9)
85	15.2 (13.2-17.1)	13.4 (11.4-16.3)	74.3 (65.6-83)	74.7 (64.2-84.2)
86	15.4 (13.4-17.3)	13.7 (11.9-16.5)	75.2 (66.4-84.1)	75.9 (65.7-85.3)

© 2026 Bijelić V et al. *JAMA Network Open*.

87	15.6 (13.7-17.5)	14.1 (12.1-16.8)	76.4 (67.4-85.3)	77.4 (67.3-87.1)
88	15.8 (13.9-17.6)	14.4 (12.3-17.1)	77.6 (68.5-86.6)	79.3 (69.5-88.9)
89	15.9 (14.1-17.8)	14.7 (12.7-17.5)	78.8 (69.7-87.9)	81.6 (71.3-90.9)
90	16.1 (14.2-17.9)	14.9 (13.1-17.8)	79.9 (70.8-89.1)	82.9 (73.1-92.9)
91	16.2 (14.3-18)	15.2 (13.4-18)	81 (71.9-90)	84.6 (75.2-94.7)
92	16.2 (14.4-18)	15.3 (13.5-18)	81.7 (72.8-90.6)	85.9 (76.9-95.9)
93	16.1 (14.3-17.9)	15.4 (13.5-17.8)	82.2 (73.6-90.7)	86.4 (77.9-97.1)
94	16 (14.2-17.8)	15.3 (13.4-17.6)	82.3 (74.1-90.5)	86.7 (78.6-97.3)
95	15.8 (14-17.6)	15.2 (13.3-17.3)	82.2 (74.2-90.3)	86.5 (79.1-96.4)
96	15.6 (13.8-17.4)	15 (13.1-17.1)	82 (73.9-90)	86.3 (78.6-95.9)
97	15.3 (13.5-17.1)	14.7 (13-16.7)	81.6 (73.3-90)	86 (77.2-95.9)
98	15.1 (13.3-16.9)	14.4 (12.7-16.2)	81.3 (72.4-90.1)	85.5 (77.1-96.3)
99	14.9 (13-16.8)	14.2 (12.3-15.9)	81 (71.5-90.4)	85.3 (76.9-97.2)
100	14.7 (12.8-16.6)	13.9 (12.1-15.8)	80.8 (70.7-90.8)	84.9 (76.1-98.1)
101	14.5 (12.6-16.5)	13.8 (12-15.7)	80.7 (70-91.4)	84.5 (75.3-99.3)
102	14.4 (12.4-16.5)	13.7 (11.9-15.6)	80.7 (69.5-92)	84.3 (74.5-99.4)
103	14.4 (12.3-16.4)	13.6 (11.7-15.4)	80.9 (69.1-92.7)	84.6 (73.8-101.2)
104	14.3 (12.3-16.4)	13.6 (11.7-15.4)	81.2 (69-93.4)	85.1 (73.7-101.1)
105	14.4 (12.3-16.4)	13.5 (11.7-15.5)	81.6 (69.1-94.2)	85.5 (73.3-100.5)
106	14.4 (12.4-16.5)	13.5 (11.5-15.5)	82.2 (69.4-95)	86.3 (72.9-100.6)
107	14.5 (12.5-16.6)	13.6 (11.6-15.5)	82.9 (69.9-95.9)	86.8 (73.2-103.1)
108	14.7 (12.6-16.7)	13.6 (11.6-15.5)	83.7 (70.6-96.7)	87.2 (74.3-104.8)
109	14.8 (12.8-16.9)	13.6 (11.6-15.6)	84.5 (71.3-97.7)	88 (74.8-105.6)
110	15 (13-17.1)	13.7 (11.6-15.6)	85.4 (72-98.7)	88.7 (75.5-107.9)
111	15.3 (13.2-17.4)	13.8 (11.5-15.7)	86.2 (72.7-99.8)	89.7 (76.9-109.7)
112	15.5 (13.3-17.6)	13.9 (11.5-15.8)	87.1 (73.5-100.7)	90.2 (78.2-109.8)
113	15.7 (13.5-17.9)	14 (11.5-15.9)	88 (74.3-101.6)	91.1 (78.8-108.7)
114	16 (13.6-18.3)	14 (11.4-16.2)	88.8 (75.1-102.4)	91.6 (79.7-108.8)
115	16.2 (13.8-18.6)	14.1 (11.3-16.6)	89.5 (75.9-103)	92.2 (80.1-110.3)
116	16.4 (13.9-18.9)	14.3 (11.2-17)	90.1 (76.6-103.6)	93.3 (80.8-111.8)
117	16.6 (14-19.3)	14.3 (11.1-17.5)	90.6 (77.1-104)	93.9 (81.7-112.8)
118	16.8 (14.1-19.6)	14.5 (11.3-18.3)	90.9 (77.4-104.4)	94.3 (83.1-114.8)

© 2026 Bijelić V et al. *JAMA Network Open*.

119	17 (14.1-19.9)	14.6 (11.3-18.8)	91 (77.5-104.6)	95.2 (83.1-116.8)
120	17.1 (14.1-20.2)	14.7 (11.3-19.2)	91 (77.3-104.6)	96.1 (82.7-117)
121	17.3 (14.1-20.5)	14.8 (11.5-19.4)	90.8 (76.9-104.6)	96.7 (82.1-117.3)
122	17.4 (14-20.7)	14.9 (11.3-19.2)	90.4 (76.3-104.5)	97.1 (82-118.7)
123	17.4 (13.9-21)	15 (11.1-19)	89.9 (75.5-104.4)	97.9 (81.1-119.8)
124	17.5 (13.8-21.3)	15 (10.9-19.4)	89.4 (74.4-104.5)	98.2 (80.8-121)
125	17.6 (13.6-21.6)	14.9 (10.6-19.9)	88.8 (73-104.6)	98.5 (81-122)
126	17.6 (13.4-21.9)	15.1 (10.5-20.2)	88.3 (71.5-105)	98.9 (78.7-122.7)
127	17.7 (13.1-22.3)	15.2 (10.3-20.5)	87.7 (69.9-105.6)	98.3 (76.8-125.7)
128	17.8 (12.9-22.8)	15.3 (10.1-21.2)	87.2 (68-106.5)	97.8 (74.8-127.3)
129	17.9 (12.5-23.3)	15.3 (9.9-21.4)	86.7 (65.8-107.6)	97.8 (72.8-129.1)
130	18.1 (12.2-24)	15.3 (9.7-22)	86.3 (63.5-109.1)	98.1 (70.6-131.7)
131	18.2 (11.7-24.7)	15.3 (9.6-23.3)	85.9 (61-110.9)	98 (67.7-134)

© 2026 Bijelić V et al. *JAMA Network Open*.

eFigure 1. Ferritin Optimal Curves for Males Aged 2 Weeks to 10 Years Estimated Using Multiple Imputation First Then Delete (MID) and Delete First Then Multiple Imputation (DMI)

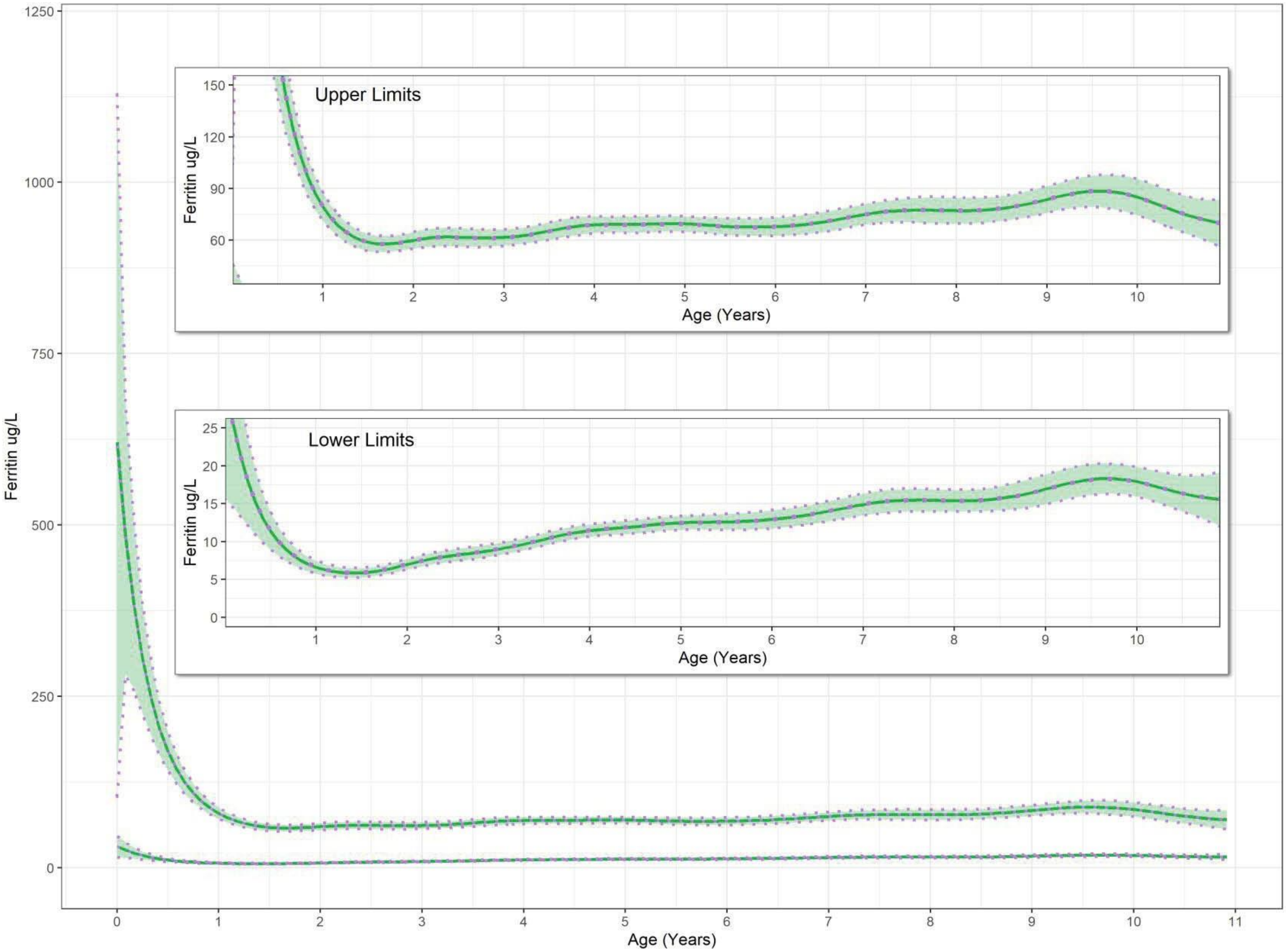

Values are estimated using two different imputation strategies: multiple imputation first then delete (MID) in green and delete then multiple imputation (DMI) in purple. The 90% CIs for the lower and upper limits of the optimal curves are presented using dotted lines. The two row inserts (row panels) show lower and upper limits separately, for better visualization.

© 2026 Bijelić V et al. *JAMA Network Open*.

eFigure 2. Ferritin Optimal Curves for Females Aged 2 Weeks to 10 Years Estimated Using Multiple Imputation First Then Delete (MID) and Delete First Then Multiple Imputation (DMI)

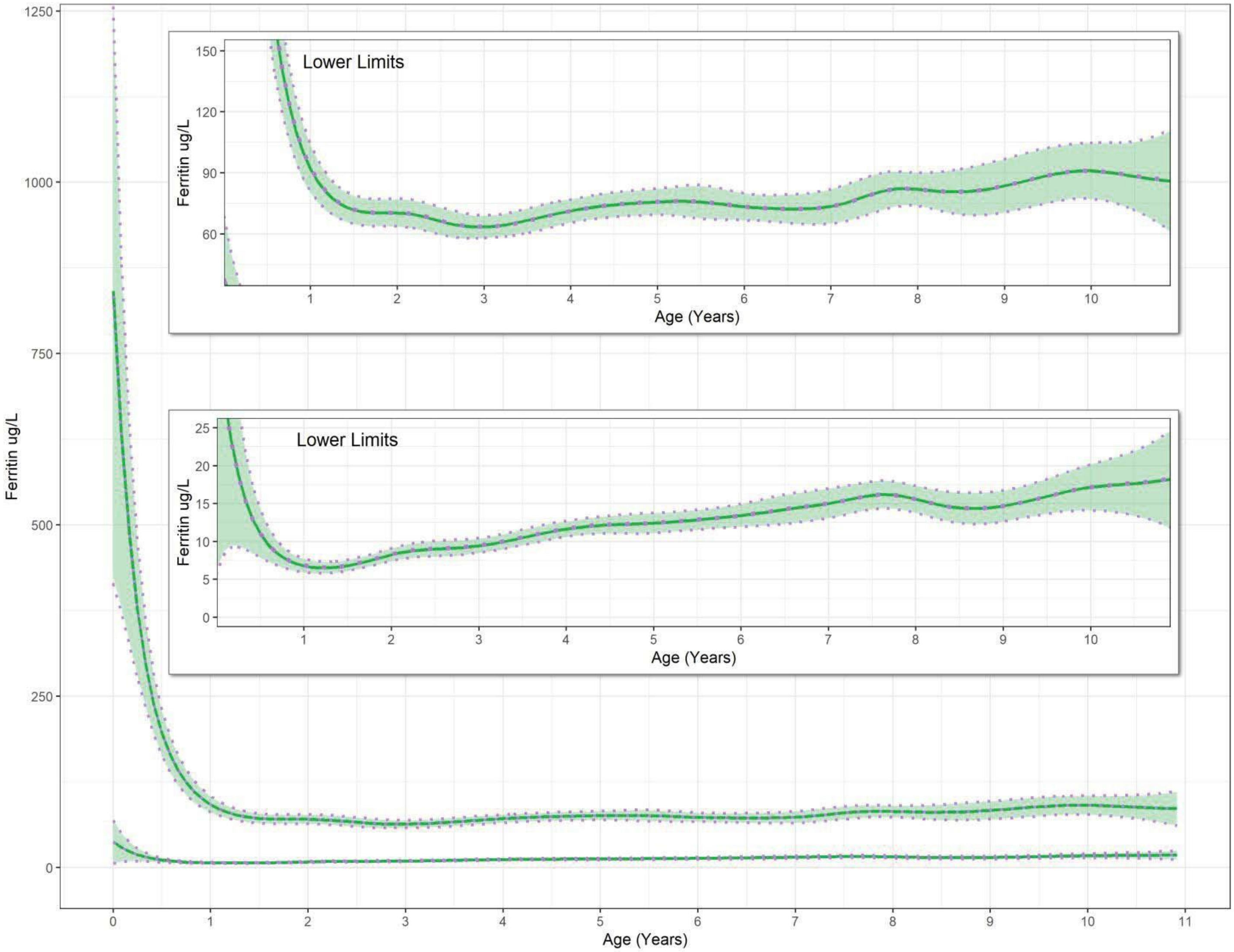

Values are estimated using two different imputation strategies: multiple imputation first then delete (MID) in green and delete first then multiple imputation (DMI) in purple with 90% confidence intervals presented using dotted lines. The wo inserts shown in the two row panels provide the lower and upper limits of the optimal curves separately.

© 2026 Bijelić V et al. *JAMA Network Open*.

eTable 3. Characteristics of Study Participants in Imputed and Complete Case Datasets Used for Optimal Curve Estimation Prior to Excluding Those Not Meeting Optimality Criteria

**Characteristics**	**All participants with ferritin values[3]** **N = 4,935**	**Participants in primary MID analysis[4]** **N = 4,935**	**Participants in DMI sensitivity analysis[5]** **N = 4,935**	**Participants in complete case sensitivity analysis[6]** **N = 3,414**
Age in months, median (IQR)	37 (18, 62)	37 (18, 62)	37 (18, 62)	36 (18, 61)
Sex, n (%)				
Female	2,322 (47.1)	2,322 (47.1)	2,322 (47.1)	1,593 (46.7)
Male	2,613 (52.9)	2,613 (52.9)	2,613 (52.9)	1,821 (53.3)
Ferritin (ug/L), median (IQR)	29 (20, 41)	29 (20, 41)	29 (20, 41)	29 (20, 41)
Missing, n (%)	0 (0)	0 (0)	0 (0)	0 (0)
CRP (mg/L), median (IQR)	0.3 (0.2, 0.6)	0.3 (0.2, 0.6)	0.3 (0.2, 0.6)	0.2 (0.2, 0.5)
Missing, n (%)	0 (0)	0 (0)	0 (0)	0 (0)
Low income cut-off (LICO), n (%)				
Above LICO	3,985 (90.5)	4,464 (90.5)	4,461 (90.4)	3,079 (90.2)
Below LICO	417 (9.5)	471 (9.5)	474 (9.6)	335 (9.8)
Missing	533 (10.8)	0 (0)	0 (0)	0 (0)
Prematurity[1] n (%)				
Not premature	4,555 (96.0)	4,734 (95.9)	4,719 (95.6)	3,248 (95.1)
Premature	189 (4.0)	201 (4.1)	216 (4.4)	166 (4.9)
Missing	191 (3.9)	0 (0)	0 (0)	0 (0)
Low birthweight[1] n (%)				
Not low birthweight	4,678 (96.3)	4,746 (96.2)	4,744 (96.1)	3,263 (95.6)
Low birthweight	181 (3.7)	189 (3.8)	191 (3.9)	151 (4.4)
Missing	76 (1.5)	0 (0)	0 (0)	0 (0)
Obese/Underweight[2] n (%)				
Not Obese/Underweight	4,447 (92.0)	4,539 (92.0)	4,540 (92.0)	3,158 (92.5)
Obese/Underweight	385 (8.0)	396 (8.0)	395 (8.0)	256 (7.5)
Missing	103 (2.1)	0 (0)	0 (0)	0 (0)
Anemia, n (%)				
No anemia	4,189 (92.5)	4,569 (92.6)	4,556 (92.3)	3,165 (92.7)
Anemia	341 (7.5)	366 (7.4)	379 (7.7)	249 (7.3)
Missing	405 (8.2)	0 (0)	0 (0)	0 (0)

[1]Applicable when ferritin measured in first 24 months of life
[2]Above or Below zBMI +/- 1.96*SD, where zBMI is BMI z-score relative to WHO reference population adjusted for age and sex
[3]Sample for reference curve estimation, also included in Table 1 of main paper
[4]Missing data for optimality criteria variables imputed with multiple imputation then deletion (MID) approach (see Methods), also included in Table 1 of main paper for primary analysis to estimate optimality curves
[5]Missing data for optimality criteria variables imputed with deletion then multiple imputation (DMI) approach (see Methods), for sensitivity analysis to estimate optimality curves

© 2026 Bijelić V et al. *JAMA Network Open*.

eFigure 3. Ferritin Optimal Curves for Females Aged 2 Weeks to 10 Years Estimated Using Multiple Imputation First Then Delete (MID) and Delete First Then Multiple Imputation (DMI)

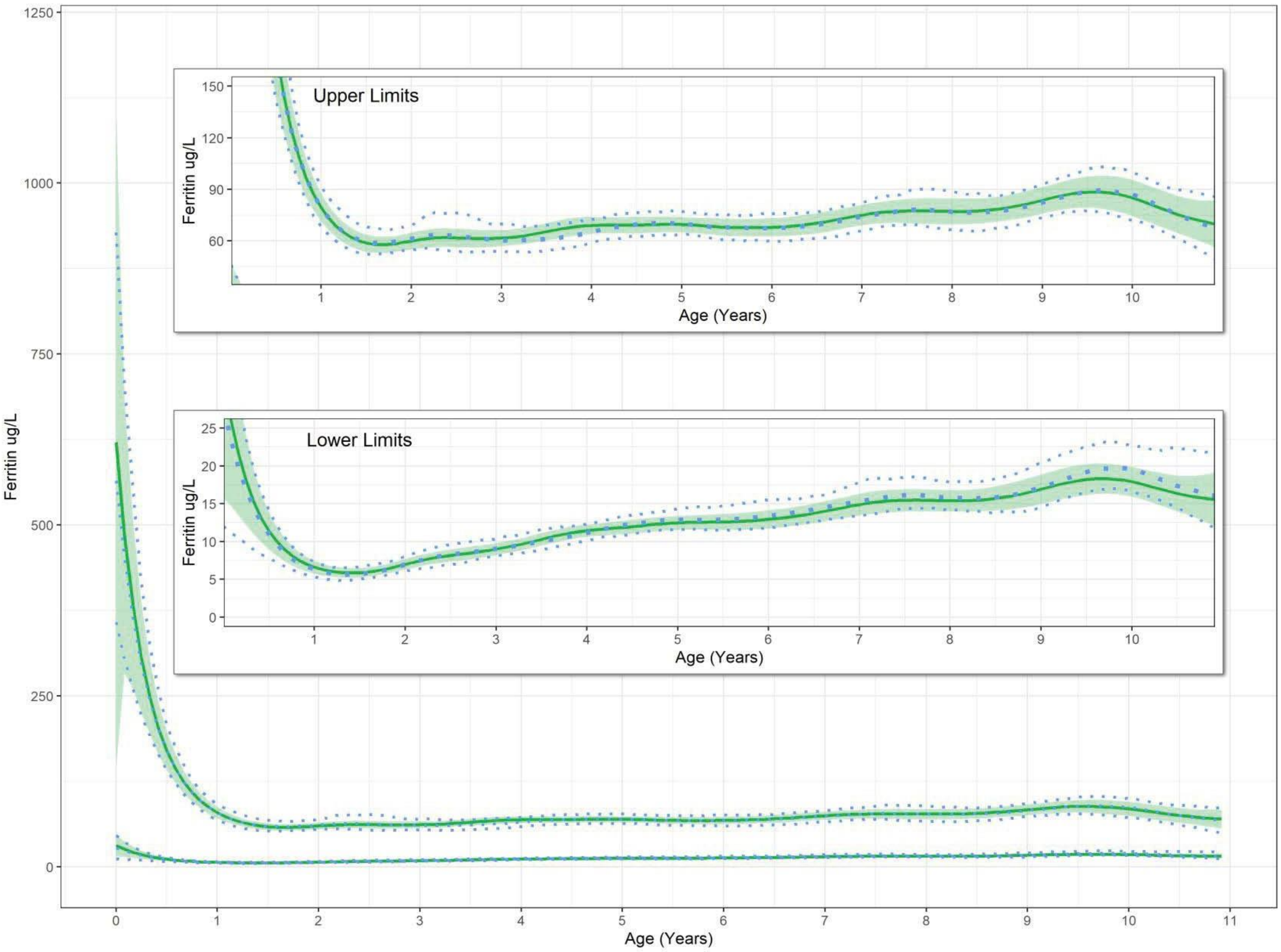

Eestimates obtained using the multiple imputation then delete (MID) approach presented in green and complete case analysis presented in sky blue. The 90% CIs are presented using dotted lines. Two inserts show lower (lower inset) and upper (upper inset) optimal curve limits magnified.

© 2026 Bijelić V et al. *JAMA Network Open*.

eFigure 4. Ferritin Optimal Curves for Females Aged 2 Weeks to 10 Years Comparing Estimates Obtained Using the Multiple Imputation Then Delete (MID) Approach and Complete Case Analysis

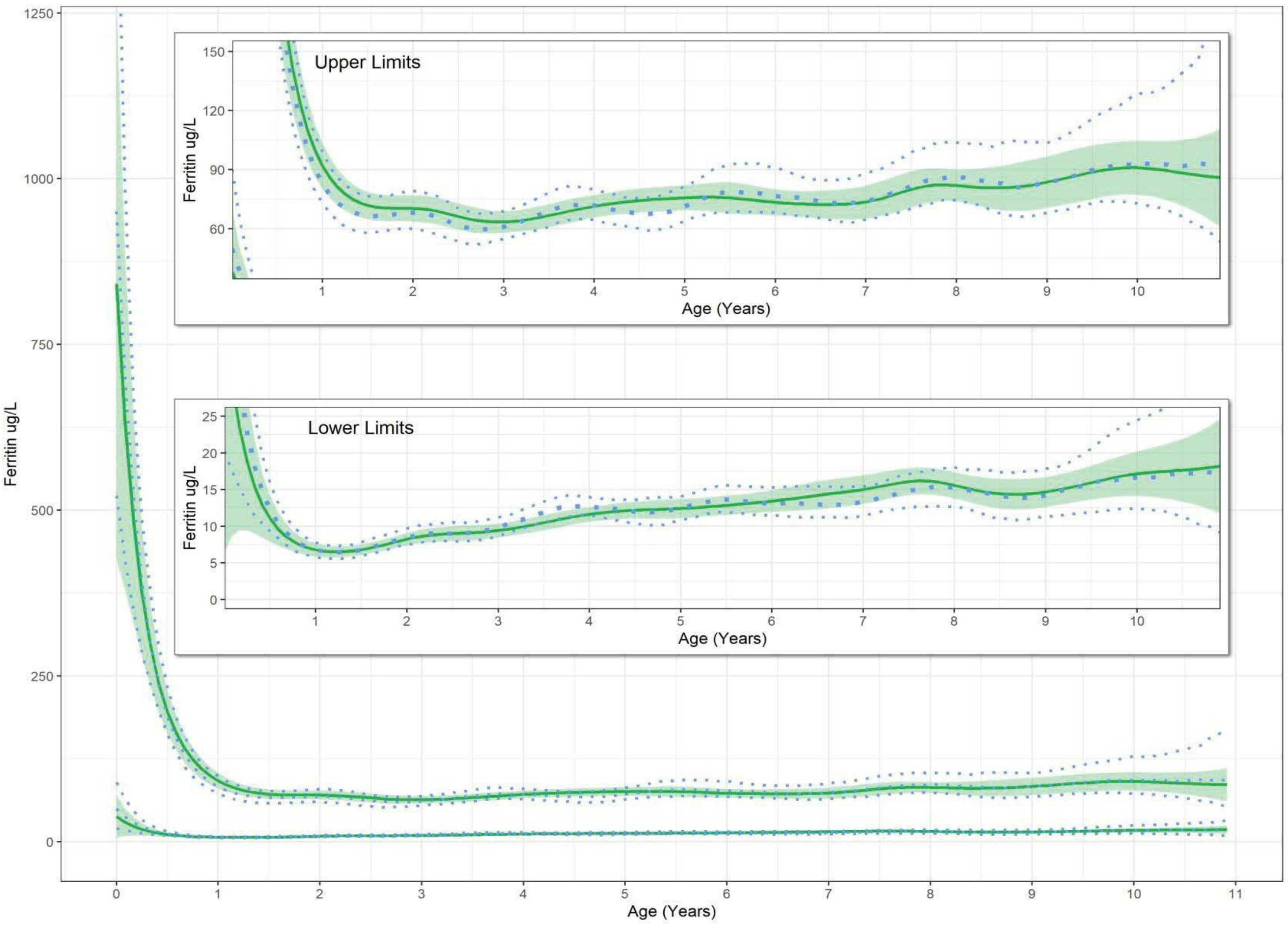

Estimates obtained using the multiple imputation then delete (MID) approach presented in green and complete case analysis presented in sky blue. The 90% CIs are presented using dotted lines. Two inserts show lower (lower inset) and upper (upper inset) optimal curve limits magnified.

© 2026 Bijelić V et al. *JAMA Network Open*.
