# Supplementary material for: Ferritin Reference Curves and Optimal Curves in Preadolescent Children
Source: JAMA Netw Open. 2026 May 15;9(5):e2613041. doi: 10.1001/jamanetworkopen.2026.13041 (PMC13179555; doi:10.1001/jamanetworkopen.2026.13041)
Supplement: Supplement 2. — Data Sharing Statement [file jamanetwopen-e2613041-s002.pdf]

Data Sharing Statement

Bijelić. Ferritin Reference Curves and Optimal Curves in Preadolescent Children. *JAMA Netw Open*. Published May 15, 2026. doi:10.1001/jamanetworkopen.2026.13041

Data
Data available: No

Additional Information
Explanation for why data not available: Please contact corresponding author for data availability.
